# Development and Validation of a Multimodal-Based Prognosis and Intervention Prediction Model for COVID-19 Patients in a Multicenter Cohort

**DOI:** 10.3390/s22135007

**Published:** 2022-07-02

**Authors:** Jeong Hoon Lee, Jong Seok Ahn, Myung Jin Chung, Yeon Joo Jeong, Jin Hwan Kim, Jae Kwang Lim, Jin Young Kim, Young Jae Kim, Jong Eun Lee, Eun Young Kim

**Affiliations:** 1 Lunit Inc., 27, Teheran-ro 2-gil, Gangnam-gu, Seoul 06241, Korea; sosal@snu.ac.kr (J.H.L.); johnahn92@lunit.io (J.S.A.); 2 Department of Radiology and Medical AI Research Center, Samsung Medical Center, Sungkyunkwan University School of Medicine, Seoul 06351, Korea; mj1.chung@samsung.com; 3 Department of Radiology, Pusan National University Hospital, Pusan National University School of Medicine and Biomedical Research Institute, Busan 49241, Korea; jeongyj@pusan.ac.kr; 4 Department of Radiology, Chungnam National University Hospital, Chungnam National University College of Medicine, Daejeon 35015, Korea; michelan@cnu.ac.kr; 5 Department of Radiology, School of Medicine, Kyungpook National University, Kyungpook National University Hospital, Daegu 41944, Korea; limjaekwang@gmail.com; 6 Department of Radiology, Keimyung University Dongsan Hospital, Keimyung University School of Medicine, Daegu 42601, Korea; jinkim0411@naver.com; 7 Department of Biomedical Engineering, Gachon University College of Medicine, Incheon 21565, Korea; youngjae@gachon.ac.kr; 8 Department of Radiology, Chonnam National University Hospital, 42 Jebong-ro, Dong-gu, Gwangju 61469, Korea; 9 Department of Radiology, Gil Medical Center, Gachon University College of Medicine, Namdong-daero 774 beon-gil, Namdong-gu, Incheon 21565, Korea

**Keywords:** COVID-19, artificial intelligence, prognosis, chest radiograph

## Abstract

The ability to accurately predict the prognosis and intervention requirements for treating highly infectious diseases, such as COVID-19, can greatly support the effective management of patients, especially in resource-limited settings. The aim of the study is to develop and validate a multimodal artificial intelligence (AI) system using clinical findings, laboratory data and AI-interpreted features of chest X-rays (CXRs), and to predict the prognosis and the required interventions for patients diagnosed with COVID-19, using multi-center data. In total, 2282 real-time reverse transcriptase polymerase chain reaction-confirmed COVID-19 patients’ initial clinical findings, laboratory data and CXRs were retrospectively collected from 13 medical centers in South Korea, between January 2020 and June 2021. The prognostic outcomes collected included intensive care unit (ICU) admission and in-hospital mortality. Intervention outcomes included the use of oxygen (O_2_) supplementation, mechanical ventilation and extracorporeal membrane oxygenation (ECMO). A deep learning algorithm detecting 10 common CXR abnormalities (DLAD-10) was used to infer the initial CXR taken. A random forest model with a quantile classifier was used to predict the prognostic and intervention outcomes, using multimodal data. The area under the receiver operating curve (AUROC) values for the single-modal model, using clinical findings, laboratory data and the outputs from DLAD-10, were 0.742 (95% confidence interval [CI], 0.696–0.788), 0.794 (0.745–0.843) and 0.770 (0.724–0.815), respectively. The AUROC of the combined model, using clinical findings, laboratory data and DLAD-10 outputs, was significantly higher at 0.854 (0.820–0.889) than that of all other models (*p* < 0.001, using DeLong’s test). In the order of importance, age, dyspnea, consolidation and fever were significant clinical variables for prediction. The most predictive DLAD-10 output was consolidation. We have shown that a multimodal AI model can improve the performance of predicting both the prognosis and intervention in COVID-19 patients, and this could assist in effective treatment and subsequent resource management. Further, image feature extraction using an established AI engine with well-defined clinical outputs, and combining them with different modes of clinical data, could be a useful way of creating an understandable multimodal prediction model.

## 1. Introduction

The coronavirus disease 2019 (COVID-19) pandemic has swept the world, and appropriate and efficient medical triaging is becoming increasingly important to prevent health services from becoming overwhelmed by excessive demand [1]. As seen in the surge of COVID-19 cases, a sudden increase in the number of infected patients can lead to a shortage of critical resources [2,3,4,5]. Effective management of such surges requires swift clinical evaluation, treatment and resource management [6]. In addition, during near-mass casualty scenarios, it is important to predict the likelihood of mortality to help prioritize treatments and resources [7,8,9]. The prognostication of disease progression in terms of required interventions and mortality are important factors to consider when treating severely ill patients [10]. Hence, the usage of artificial intelligence (AI) in prognosis prediction is growing and the research interests are even expanding into areas such as COVID-19 prognosis prediction in telehealthcare [11,12].

Chest X-ray (CXR) is a first-line imaging modality that allows rapid, affordable and accessible chest imaging worldwide. CXR is a useful medical triage resource for patients with a high pre-test probability of COVID-19 and who are suspected to have moderate-to-severe disease [13]. However, for COVID-19 patients, the interpretation of CXR varies greatly between readers because of the differences in the skillset of the readers and the added difficulty in interpretation due to the severe changes in the CXRs from COVID-19 [14,15]. The efforts to reduce the variation and improve the quality of interpretation have resulted in the use of AI with imaging to grow in the context of COVID-19. It has been shown that AI can help interpret CXRs effectively in COVID-19 patients [16]. Khuzani et al. showed that a CXR AI classifier trained on 420 images can accurately classify, with 94% accuracy, the CXR images into normal, COVID-19 and non-COVID-19 pneumonia, based on imaging findings alone [17]. Naturally, combining different modes of data used in clinical medicine to predict the events in COVID-19 patients has become the goal of many researchers. For example, a previous model created in the US, which was trained on a cohort of 1834 patients from a single institution, showed that multimodal AI models using combined CXR and clinical data were more useful than single-modal models for predicting the risk of critical illness progression in patients with COVID-19 [18]. However, there is a lack of evidence for creating a model that mimics the way that clinicians use information to derive the necessary management and the prognosis in COVID-19 patients.

The aim of this study was to develop and validate models for predicting adverse events in COVID-19 patients using a multimodal approach based on clinical findings, laboratory data, and AI-interpreted features of CXRs in a nationwide multicenter cohort.

## 2. Related Works

Our study has several differences compared to earlier studies. Previous studies focused either on diagnostic or severity prediction, and did not take into account the next decision for the patient, which is a necessary intervention for treatment [19,20,21,22,23]. In our study, the model was trained with both prognostic outcomes and required interventions, such as O_2_ supplementation and intensive care unit (ICU) admission. Being able to predict the necessary treatment resources is especially important in managing highly infectious diseases that cause a surge in cases. To the author’s knowledge, this is the first multimodal model that utilizes three different modes of data that are clinically relevant and enables the prediction of both prognosis and treatment.

Furthermore, previous works on using medical imaging for prognosis prediction used end-to-end models that utilized convolutional neural networks or radionics features [18,20,21]. By contrast, we used an established AI engine for image feature extraction from the CXRs, which can provide clinically interpretable and relevant information in a hospital setting. These imaging features are clinically well-defined findings, routinely used by clinicians. This allows users to be able to infer the logic behind the predicted prognosis and treatments heuristically, which is useful for understanding AI behavior.

## 3. Materials and Methods

### 3.1. Study Population

This study used the data repository of the Korean Imaging Cohort for COVID-19 (KICC-19), which is the multicenter data from 13 main referral institutions across South Korea. These data were created by Korean Society of Thoracic Radiology (KSTR) members in 2020 [24]. All of the enrolled patients’ data were anonymized and collected on a cloud-based data storage platform. This study included consecutive patients from the data repository between January 2020 and June 2021. The inclusion criteria were consecutive adult patients (≥18 years old) with real-time reverse transcriptase polymerase chain reaction (RT-PCR)-proven COVID-19, who underwent a diagnostic full inspiratory of the CXR at the participating institution. Patients were excluded if no baseline data were available, or if no details of the clinical course were recorded.

This retrospective study was approved by the institutional review board (GCIRB2021-312), which waived the requirement for informed consent due to the retrospective nature of data collection and the use of anonymized images.

### 3.2. Data Collection and Study Definition

The clinical findings, CXR and laboratory data of each patient, obtained at admission, were collected from the cloud-based data storage platform. The initial CXR of each patient, obtained in anteroposterior or posterior–anterior views, was collected. The clinical findings included demographics (age and sex), comorbidities (hypertension, diabetes, cardiovascular disease and history of cancer) and clinical symptoms (fever, cough, sputum, myalgia, dyspnea and sore throat). The initial laboratory data included lymphocyte and thrombocyte counts and C-reactive protein (CRP) and lactate dehydrogenase (LDH) levels. Lymphocytopenia was defined as a lymphocyte count of less than 1500 cells/µL. Thrombocytopenia was defined as a platelet count of less than 150,000 cells/µL. The predefined clinical thresholds for LDH and CRP elevation were 50 mg/L and 250 U/L, respectively. The clinical outcomes included the requirement for O_2_ supplementation, mechanical ventilation, extracorporeal membrane oxygenation (ECMO) and ICU admission, as well as in-hospital mortality. In-hospital mortality was defined as death resulting from clinically compatible illnesses in COVID-19 patients during hospitalization.

### 3.3. Dataset Partitioning for Multicenter Validation

To develop and validate the COVID-19 prognosis and intervention prediction model, the data from 13 medical centers were divided into model development and performance validation datasets. The smallest unit of division was one medical center. For this nationwide multicenter cohort study, various levels of care centers with different medical roles were included. Random data partitioning by mixing all the data can omit the characteristics of each center and impair sample representativeness [25]. Therefore, we performed center-based semi-random data partitioning, only considering the balance of the number of clinical events between development and validation sets.

### 3.4. Image Feature Extraction from CXR

A commercially available AI model, a deep learning algorithm that detects 10 common CXR abnormalities (Lunit INSIGHT for Chest Radiography Version 3.1.3.5; Lunit, Seoul, South Korea) [26], was used to infer imaging features from the initial CXR images. The DLAD-10 has been trained on over 168,000 CXR images annotated by 20 board-certified radiologists. The DLAD-10 is a ResNet-34-based algorithm that uses a CXR DICOM file as an input, without needing further image processing. Subsequently, the DLAD-10 outputs were used as inputs to the prediction model. The DLAD-10 outputs used were nodules, consolidation, pneumothorax, pleural effusion, cardiomegaly, nodule, pneumoperitoneum, mediastinal widening, calcification and atelectasis. Each output had a value between 0 and 100, and this represents the probability of the presence of lesions. This output was used without any calibration.

### 3.5. Prognosis Prediction Model Development

**Figure 1:**
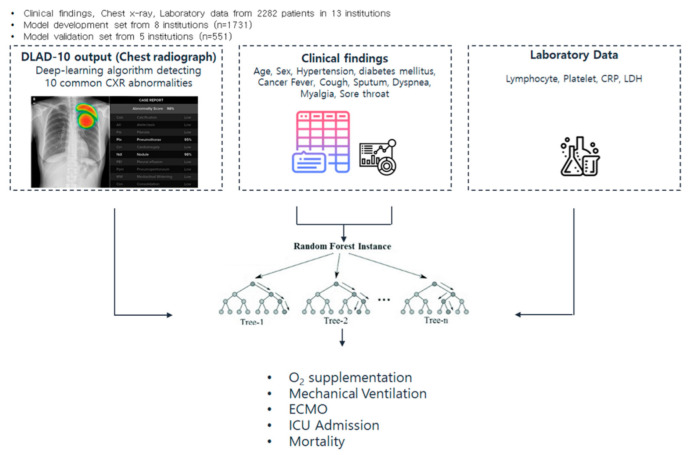
Schematic of the study workflow and performance of risk prediction model.

Prognosis prediction models were trained to predict O_2_ supplementation, mechanical ventilation, ECMO, ICU admission, in-hospital mortality and all adverse events. All adverse events were defined as having any one of the event outcomes. A random forest model with a quantile classifier was used to predict events from CXR, laboratory data and clinical information using Fast unified random forests for survival, regression and classification (RF-SRC), and non-parametric statistical estimation [27]. For model training, out-of-bag (OOB) samples were used as test samples. Feature selection was performed using the mean decrease in accuracy and mean decrease in Gini, as suggested by Han et al. [28], and a maximum of five iterations were performed. The importance of the features was defined as the mean decrease in forest’s performance for the randomly permuted OOB samples. The workflow schemes for the data configuration, data type and model learning are shown in Figure 1.

### 3.6. Statistical Analysis

The statistical analysis of the differences between the development and validation sets was performed using Pearson’s chi-square test or Fisher’s exact test for categorical variables and an independent t-test for continuous variables. To compare the prognosis prediction model, the area under the receiver operating curve (AUROC) was calculated for binary clinical event classification and AUROCs were compared using the DeLong test in the pROC R package (version 3.6.3; R Project for Statistical Computing, https://www.r-project.org, accessed on 1 January 2022) [29,30]. The statistical significance was set at *p* < 0.05.

## 4. Results

### 4.1. Patient Characteristics

The patient characteristics of the overall, training and validation sets are presented in Table 1. A total of 2282 patients who met the inclusion criteria were evaluated across the datasets. The mean age of patients was 52.8 years (interquartile range, 40–70 years); 1193 were male (52.3%) and 1089 were female. Of the 2282 patients from 13 centers, 1731 patients from 8 centers were included in the development set and 551 patients from 5 centers were included in the test set. The mean age was higher in the training set (53.5 ± 20.4) than in the validation set (50.6 ± 17.9) (*p* = 0.003). The proportion of women was higher in the training set (859/1731 [49.6%]) than in the validation set (230/551 [41.7%]) (*p* = 0.001). The proportion of symptomatic patients was higher in the training set (477/551 [86.6%]) than in the validation set (1246/1731 [72%]) (*p* < 0.001). The proportion of patients with elevated LDH was significantly higher in the validation set (449/551 [90.5%]) than in the training set (603/1731 [42.8%]) (*p* < 0.001). The proportion of ICU admissions was higher in the validation set (50/551 [9.1%]) than in the training set (74/1731 [4.3%]) (*p* < 0.001). The detailed composition of the patient characteristics in each center is summarized in Supplementary Table S1.

### 4.2. Performance of Adverse Events Prediction Model

**Figure 2:**
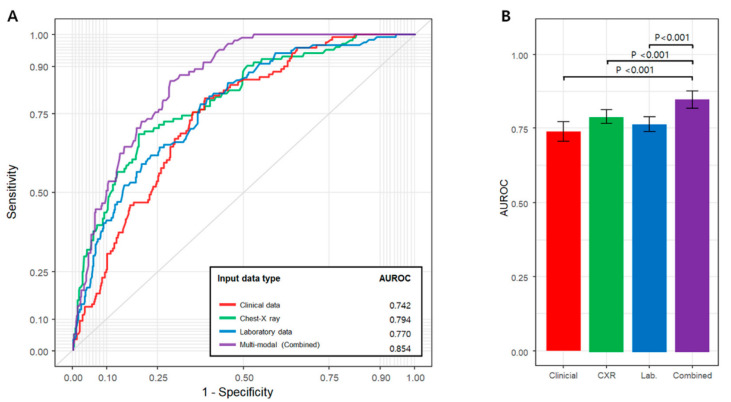
(**A**) Average receiver operating characteristic (ROC) curves under conditions: unimodal model using clinical data, chest X-ray, and laboratory data and multimodal model. (**B**) Area under the ROC (AUROC) shows combined model has superior performance than others.

The prediction performances of the models for the three data types and the multimodal model are shown in Table 2. The clinical finding-based model had an AUROC of 0.742 (95% confidence interval [CI], 0.696–0.788) for predicting all adverse events. The laboratory data-based model had an AUROC of 0.794 (0.745–0.843) for predicting all adverse events. The CXR-based model had an AUROC of 0.770 (0.724–0.815) for predicting all adverse events. The multimodal model that combined all three data had an AUROC of 0.854 (0.820–0.889) for predicting all adverse events, which was significantly higher than that of each single-modal model (Figure 2) (all Ps < 0.001). The multimodal model showed the best performance in all remaining events, except for predicting ECMO use, which had the smallest number of patients (11 patients in five centers). The average AUROC of the models predicting all adverse events was higher in the order of CXR, laboratory data and clinical data, but there was no significant difference.

### 4.3. Feature Importance Analysis

**Figure 3:**
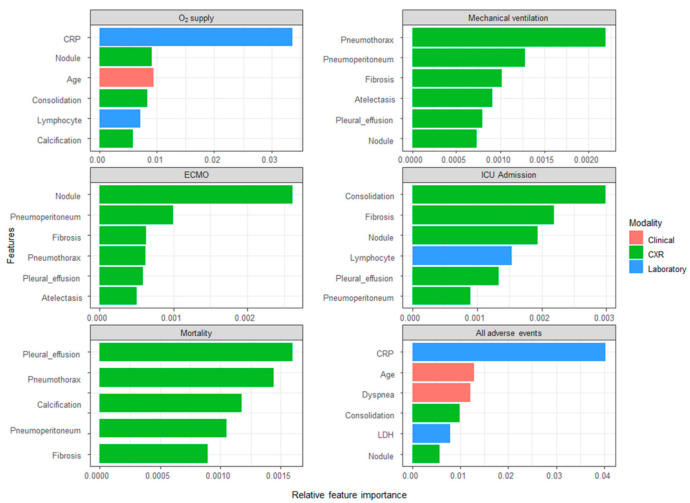
Importance of relative features to predict events.

Based on the multimodal model, the relative feature importance of the model learned for all events is shown (Figure 3). The red, green and blue bars represent the clinical data, the DLAD-10 outputs and laboratory findings, respectively. The CRP level was the most important predictor of O_2_ supplementation. In predicting mechanical ventilation, the DLAD-10 outputs were selected as the top five important features. This pattern was similar for predicting ECMO and in-hospital mortality. Consolidation was the most important feature for predicting ICU admission. In predicting all adverse events, a mix of different modes of data was deemed important. The most important feature was CRP in the laboratory data, followed by age, dyspnea in the clinical data and consolidation of the DLAD-10 outputs.

## 5. Discussion

In this study, we developed a multimodal-based predictive model by combining AI-interpreted CXR outputs, clinical findings and laboratory data to predict adverse outcomes in patients with COVID-19. As revealed in our study, (1) in the external validation set, the performance of the AI-interpreted CXR-based predictive model had an AUROC of 0.7 or higher for the prediction of major adverse events, which included O_2_ supplementation, mechanical ventilation, ECMO, ICU admission and in-hospital mortality, and (2) the multimodal prediction model using a combination of AI-interpreted CXR outputs, clinical findings and laboratory data generally had better predictive performance for major adverse events.

Combining clinical, laboratory and imaging findings is fundamentally how clinicians derive differential diagnoses and proceed with an appropriate treatment [31]. Specific clinical symptoms and signs of COVID-19, such as fever and sore throat, and CXR findings, such as the pattern of consolidation, help clinicians to determine the likelihood of diagnosis, required treatment and likely prognosis [32,33]. Therefore, the multimodal interpretation of data is an important consideration when developing AI prediction tools. It was shown in our study that the performance of the prediction model incrementally increased when using imaging, clinical findings and laboratory data together, compared to when each data modality was used alone. There has been previous work on the usage of multimodal data in telehealthcare settings [34], but we have focused on the clinical information that is routinely collected by the clinical team at medical institutions.

We have developed and validated a COVID-19 prognosis and intervention prediction model using a two-step multimodal approach based on clinical findings, laboratory data, and AI-interpreted CXR features in a nationwide cohort enrolled from 13 centers. This is one of the first studies to examine multicenter data split by each institution. Previous studies have validated data split at the patient level, rather than at the institutional level. Each center differs vastly in characteristics, such as patient demographics, treatment resources available and imaging equipment vendors. By separating the training and validation data from each center, we were able to show that our multimodal approach achieved high performance and generalized well to data that are characteristically different from the training dataset and have not been seen before. It has been shown that previous imaging-based AI models for COVID-19 prognostication may be inadequate for real clinical usage [35,36]. The major reason for this was the lack of external validation data. By separating the training and validation datasets by each center, we are able to keep each dataset independent from one another and maintain the sample representativeness.

There are several advantages of using a commercially available AI engine for image feature extraction. The biggest benefit is that AI engine performance has been proven via validations in multiple independent peer-reviewed studies [37,38,39,40,41]. The DLAD-10 has previously shown to have good performance in detecting the 10 clinically well-defined and important abnormalities, with AUROCS ranging 0.895–1.00 in CT-confirmed datasets [26]. Furthermore, the engine has shown good performance in COVID-19 patients [42,43,44]. The performance of the DLAD-10 is high because it has been trained and validated on large datasets, utilizing over three million images for pretraining and fine-tuning. The quantity of the data is unparalleled compared with laboratory-created AI engines, which are often used only for research purposes. Similar prognostication studies have used approximately 2000 CXR images for training, validation, and internal and external testing [28]. Finally, commercially available products undergo rigorous review and validation before they are approved for clinical use [45]. The model had both CE and KFDA markings for clinical use.

### 5.1. Benefits of Using a Feature Extractor (DLAD-10) with Clinically Defined Outputs

The outputs of the DLAD-10 are clinically important and have well-defined classifications. Using the classifications actively used by clinicians when making clinical decisions, rather than incomprehensible AI interpretations of CXR in a numerical format, could enable clinicians to heuristically understand and have confidence in the model’s prediction. As an example, the DLAD-10 was trained on COVID-19 pneumonia data and the COVID-19 pneumonia-related changes were output as consolidation. This, together with pre-existing medical knowledge about the presence of consolidation on the CXRs of patients with COVID-19, helps clinicians understand why the correlation with consolidation output is the strongest for both prognosis and treatment prediction [46]. Without understandable AI model classifications of CXRs, clinicians are unable to understand how the model arrived at the prediction. The ability to extract understandable and clinically meaningful features from images could be an important consideration when making prediction models, to increase the understandability of the model and improve acceptance in clinical use [47].

### 5.2. Benefits of Using the Two-Step Ensemble Approach with Imaging Extractor

The two-step ensemble model implemented in this study had several advantages. The ensemble approach with an imaging feature extractor enables clinicians to have a good understanding of how each variable, including imaging features, affects prognosis and required treatments. This explainable AI allows clinicians to understand the manner in which the prediction model behaves and the relative importance of each input.

Knowledge of the relative importance of each variable enables clinicians to be wary of the appropriate treatments required, as well as the likely prognosis when a patient first presents to them. This helps in planning treatment and managing resources ahead [6]. An understandable model would allow such a thought process to occur, even without using the prediction model every time the patient is seen. For instance, if an elderly patient presents with fever, cough and consolidation on CXR, clinicians will be able to think that the patient is more likely to require an ECMO machine, twice as much as a patient with similar symptoms and signs but with fibrosis on CXR.

Knowing the relative importance of variables can help to better understand the disease process. This is important for emerging diseases such as COVID-19 or diseases that are not fully understood. By looking at the impact of each clinical variable, clinicians can form hypotheses and research questions, ultimately enabling understanding of the disease pathology and appropriate treatments, using large amounts of data and machine learning. In addition, the fact that we extracted medically well-known radiographical features from a CXR allows clinicians to form a logical hypothesis regarding COVID-19 and investigate further. For instance, it is now established that CXR consolidation can be unusually severe in COVID-19, and this does not correlate well with the clinical presentation [46]. Using this model earlier during the pandemic could have helped to discover this particular knowledge sooner. The ensemble approach can be utilized in other prediction models, and its potential use is not limited to COVID-19.

Finally, although predicting mortality has been explored in COVID-19-related studies, this study is one of the first that aimed to predict the interventions required for treating severe respiratory conditions, including ECMO, O_2_ supplementation and mechanical ventilation. Knowing the required interventions and prognosis is crucial, as appropriate and early interventions have been shown to be effective in improving COVID-19 prognosis [48,49]. Furthermore, ICU beds and ECMO are critical resources that are short in number, especially in developing countries. As seen in the oxygen shortage crisis during the pandemic, it is important to be able to predict the demand for each intervention based on the patient’s presentation.

This study had several limitations. First, the training and validation datasets were not completely balanced in terms of the predicted outcomes. However, this was because the data split was performed by each center. We believe that our approach considers the real world, where hospitals differ in the resources and equipment available. For instance, not all hospitals have multiple ECMO machines available, which would have affected the number of patients treated with ECMO. For more common interventions, such as O_2_ supplementation, the variation was less obvious. Second, we only used the initial CXR at admission for each institution. In the future, it would be better to conduct an analysis using serial CXRs taken during hospitalization. Third, we did not consider the sequential or temporal relationship between clinical data and outcomes. In a future study, we could focus on developing models that can predict the timeframe until a clinically relevant event or intervention.

## 6. Conclusions

We utilized the commercially available DLAD-10 to extract the image features and combined them with a variety of clinical data to help predict the prognosis and required treatments. Our goal was to create a model that mimics the actual clinical thought process, through which clinicians review the CXR, classify the findings, and combine this information with other clinically relevant information to derive the necessary treatments and the likely prognosis. This understandable AI approach may enable efficient and timely treatment for patients and better resource management during the COVID-19 outbreak.

## Figures and Tables

**Table 1:** Baseline characteristics of patients from all centers.

Characteristics	Overall (N = 2282)	Development (N = 1731)	Validation (N = 551)	*p* Value
Age (years)	52.8 ± 19.8	53.5 ± 20.4	50.6 ± 17.9	0.003
Sex				0.001
Male	1193 (52.3)	872 (50.4)	321 (58.3)	
Female	1089 (47.7)	859 (49.6)	230 (41.7)	
Comorbidity				
Any comorbidities	942 (41.3)	718 (41.5)	224 (40.7)	0.732
Hypertension	690 (30.2)	531 (30.7)	159 (28.9)	0.418
Diabetes	419 (18.4)	319 (18.4)	100 (18.1)	0.883
Cardiovascular disease	135 (5.9)	104 (6.0)	31 (5.6)	0.741
History of cancer	115 (5)	81 (4.7)	34 (6.2)	0.163
Symptoms				
Any symptoms	1723 (75.5)	1246 (72.0)	477 (86.6)	<0.001
Fever	919 (40.3)	634 (36.6)	285 (51.7)	<0.001
Cough	995 (43.6)	699 (40.4)	296 (53.7)	<0.001
Sputum	653 (28.6)	435 (25.1)	218 (39.6)	<0.001
Dyspnea	404 (17.7)	276 (15.9)	128 (23.2)	<0.001
Myalgia	550 (24.1)	344 (19.9)	206 (37.4)	<0.001
Sore throat	396 (17.4)	264 (15.3)	132 (24.0)	<0.001
Initial laboratory findings				
Lymphocyte count < 1000/μL *	615 (29.7)	459 (30.1)	156 (28.6)	0.503
Platelet count < 150,000/μL *	388 (18.7)	284 (18.6)	104 (19.0)	0.826
LDH > 300 U/L *	1052 (55.2)	603 (42.8)	449 (90.5)	<0.001
CRP > 50 mg/L *	471 (23.1)	345 (22.9)	126 (23.5)	0.783
Clinical outcomes				
O_2_ supplementation	408 (17.9)	323 (18.7)	85 (15.4)	0.085
Mechanical ventilation	117 (5.1)	84 (4.9)	33 (6.0)	0.292
ECMO	32 (1.4)	21 (1.2)	11 (2.0)	0.173
ICU admission	124 (5.4)	74 (4.3)	50 (9.1)	<0.001
In-hospital mortality	106 (4.6)	85 (4.9)	21 (3.8)	0.286

ECMO, extracorporeal membrane oxygenation; ICU, intensive care unit. Values in parentheses are percentages. Values are presented as mean ± standard deviation, where applicable. * Lymphocytes, platelets, LDH and CRP results were available for 2071, 2075, 1902 and 2041 patients, respectively.

**Table 2:** Predictive performance of the models in external validation.

Adverse Event Type	Area under the ROC Curve			
	Clinical Findings	Laboratory Data	CXR	Combined
O_2_ supplementation	0.753 (0.703–0.802)	0.757 (0.708–0.806)	0.701 (0.648–0.754)	0.812 (0.772–0.852)
Mechanical ventilation	0.735 (0.646–0.825)	0.852 (0.780–0.923)	0.807 (0.726–0.888)	0.880 (0.810–0.950)
ECMO	0.664 (0.489–0.839)	0.794 (0.627–0.960)	0.650 (0.525–0.776)	0.745 (0.611–0.879)
ICU admission	0.708 (0.633–0.782)	0.711 (0.607–0.815)	0.784 (0.711–0.856)	0.838 (0.770–0.906)
In-hospital mortality	0.762 (0.655–0.869)	0.805 (0.700–0.910)	0.838 (0.757–0.919)	0.877 (0.792–0.962)
All adverse events	0.742 (0.696–0.788)	0.794 (0.745–0.843)	0.770 (0.724–0.815)	0.854 (0.820–0.889)

ROC, receiver operating characteristic; CXR, chest radiograph; ECMO, extracorporeal membrane oxygenation; ICU, intensive care unit.

## Data Availability

The data presented in this study are available on request from the corresponding author.

## Supplementary Materials

The following supporting information can be downloaded at: https://www.mdpi.com/article/10.3390/s22135007/s1. Table S1. Baseline characteristics of patients from each center.
