# Supplementary material for: Development and Validation of a Multimodal-Based Prognosis and Intervention Prediction Model for COVID-19 Patients in a Multicenter Cohort
Source: Sensors (Basel). 2022 Jul 2;22(13):5007. doi: 10.3390/s22135007 (PMC9269794; doi:10.3390/s22135007)
Supplement: Supplementary file 1 [file sensors-22-05007-s001.zip › sensors-1775272-supplementary.pdf]

Table S1. Baseline characteristics of patients from each center.

Characteristics	Development Set								Validation Set				
	**Center 2 (N = 275)**	**Center 3 (N = 131)**	**Center 6 (N = 162)**	**Center 7 (N = 293)**	**Center 10 (N = 377)**	**Center 11 (N = 227)**	**Center 12 (N = 129)**	**Center 13 (N = 137)**	**Center 1 (N = 16)**	**Center 4 (N = 50)**	**Center 5 (N = 377)**	**Center 8 (N = 40)**	**Center 9 (N = 68)**
**Age (years)**	50.3 ± 16.1	66.9 ± 19.3	62.3 ± 15.2	63.4 ± 20.1	47.8 ± 18.3	36.3 ± 19.4	55.0 ± 17.2	58.4 ± 18.2	55.6 ± 19.0	47.4 ± 17.9	48.9 ± 17.1	48.3 ± 17.8	62.7 ± 17.2
**Sex**													
Male	134 (48.7)	55 (42.0)	92 (56.8)	159 (54.3)	184 (48.8)	117 (51.5)	72 (55.8)	59 (43.1)	10 (62.5)	25 (50.0)	231 (61.3)	17 (42.5)	38 (55.9)
Female	141 (51.3)	76 (58.0)	70 (43.2)	134 (45.7)	193 (51.2)	110 (48.5)	57 (44.2)	78 (56.9)	6 (37.5)	25 (50.0)	146 (38.7)	23 (57.5)	30 (44.1)
**Any comorbidity**													
No	167 (60.7)	49 (37.4)	75 (46.3)	138 (47.1)	257 (68.2)	181 (79.7)	66 (51.2)	80 (58.4)	8 (50.0)	33 (66.0)	248 (65.8)	18 (45.0)	20 (29.4)
Yes	108 (39.3)	82 (62.6)	87 (53.7)	155 (52.9)	120 (31.8)	46 (20.3)	63 (48.8)	57 (41.6)	8 (50.0)	17 (34.0)	129 (34.2)	22 (55.0)	48 (70.6)
**Any symptoms**													
No	61 (22.2)	66 (50.4)	62 (38.3)	77 (26.3)	134 (35.5)	50 (22.0)	9 (7.0)	26 (19.0)	4 (25.0)	13 (26.0)	45 (11.9)	6 (15.0)	6 (8.8)
Yes	214 (77.8)	65 (49.6)	100 (61.7)	216 (73.7)	243 (64.5)	177 (78.0)	120 (93.0)	111 (81.0)	12 (75.0)	37 (74.0)	332 (88.1)	34 (85.0)	62 (91.2)
**Clinical outcomes**													
O_2 supplementation	49 (17.8)	34 (26.0)	21 (13.0)	75 (25.6)	64 (17.0)	27 (11.9)	31 (24.0)	22 (16.1)	1 (6.3)	0 (0.0)	50 (13.3)	11 (27.5)	23 (33.8)
Mechanical ventilation	22 (8.0)	6 (4.6)	13 (8.0)	18 (6.1)	0 (0.0)	3 (1.3)	12 (9.3)	10 (7.3)	0 (0.0)	6 (12.0)	12 (3.2)	4 (10.0)	11 (16.2)
ECMO	6 (2.2)	2 (1.5)	3 (1.9)	0 (0.0)	0 (0.0)	1 (0.4)	7 (5.4)	2 (1.5)	0 (0.0)	0 (0.0)	5 (1.3)	0 (0.0)	6 (8.8)
ICU admission	4 (1.5)	8 (6.5)	4 (2.5)	8 (2.7)	4 (1.1)	3 (1.3)	34 (26.4)	9 (6.6)	0 (0.0)	7 (5.6)	24 (6.4)	9 (22.5)	10 (14.7)
In-hospital mortality	4 (1.5)	10 (7.6)	14 (8.6)	23 (7.8)	0 (0.0)	2 (0.9)	20 (15.5)	12 (8.8)	0 (0.0)	0 (0.0)	13 (3.4)	1 (2.5)	7 (10.3)
All adverse events	51 (18.5)	38 (29.0)	35 (21.6)	100 (34.1)	66 (17.5)	27 (11.9)	59 (45.7)	34 (24.8)	1 (6.3)	7 (14.0)	60 (15.9)	16 (40.0)	34 (50.0)

ECMO, extracorporeal membrane oxygenation, ICU = intensive care unit.
